# Effects of Repeated Intravenous Injections of Autologous Adipose-Derived Mesenchymal Stromal Cells Expressing an Allogeneic MHC Protein in a Mouse Model of Diabetic Nephropathy

**DOI:** 10.3390/cells15020196

**Published:** 2026-01-20

**Authors:** Fuxuan Li, Liangyu Zhao, Shengkun Wang, Ruixue Chen, Meiqi Meng, Yan Fu, Lin Wei, Wei Liu, Huixian Cui, Jun Ma, Matthew D. Griffin, Cuiqing Ma

**Affiliations:** 1 Key Laboratory of Immune Mechanism and Intervention on Serious Disease in Hebei Province, Department of Immunology, Hebei Medical University, Shijiazhuang 050017, Chinaliuweihebmu@hebmu.edu.cn (W.L.); 2 Hebei Medical University-National University of Ireland Galway Stem Cell Research Center, Hebei Medical University, Shijiazhuang 050017, China; 3 Core Facilities and Centers, Hebei Medical University, 361 East Zhongshan Road, Shijiazhuang 050017, China; 4 Regenerative Medicine Institute (REMEDI) at CÚRAM Research Ireland Centre for Medical Devices, School of Medicine, University of Galway, H91 TK33 Galway, Ireland

**Keywords:** diabetes, mesenchymal stromal cells, adipose-derived mesenchymal stromal cells, diabetic nephropathy, allogeneic cell therapies, immune responses, inflammation, regulatory T cells

## Abstract

**Highlights:**

**What are the main findings?**

- In a mouse model of diabetes, two sequential intravenous injections of adipose-derived mesenchymal stromal cells (ADSCs) modified to express an allogeneic class I major histocompatibility complex (MHC I) protein ameliorated diabetic nephropathy more effectively than autologous ADSCs.
- The beneficial effects of allogeneic MHC I-expressing ADSCs were associated with reduced renal inflammation and fibrosis and increased regulatory T cell infiltration.
- Allogeneic MHC I-expressing ADSCs were, however, also associated with development of IgG allo-antibodies and liver inflammation.

**What are the implications of the main findings?**

- In diabetic nephropathy, allogeneic mesenchymal stromal cells (Allo-MSCs) have potential for both beneficial and harmful immunological responses following repeated intravenous injections.
- Further investigation of the mechanisms underlying immune responses to allogeneic MSCs may guide the development of safer and more effective cell therapies for diabetic complications.

**Abstract:**

Diabetic nephropathy (DN) is the most common cause of kidney failure worldwide. Mesenchymal stromal cells (MSCs) have demonstrated promise for treating DN by promoting kidney repair and regulating inflammation. Allogeneic (Allo)-MSCs may have similar or superior anti-inflammatory effects to autologous (Auto)-MSCs but also have potential to elicit adverse immune responses due to major histocompatibility complex (MHC) mismatches. To better understand how MSC-delivered allo-antigens influence therapeutic effects of Allo-MSCs compared to Auto-MSCs in DN, lentiviral transduction was used to generate adipose-derived MSCs (ADSCs) from DBA/2J (H-2^d^) mice which expressed an allogeneic class I MHC protein (H-2K^b^). H-2K^b^-ADSCs were injected intravenously into male DBA/2J mice at 11 and 13 weeks after initiation of diabetes, and their effects on renal functional and structural indices were compared at week 15 with those of diabetic DBA/2J recipients of vehicle alone or of empty vector-transduced DBA/2J ADSCs (EV-ADSCs). Both EV-ADSCs and H-2K^b^-ADSCs resulted in reduced kidney/total body weight ratio, blood urea nitrogen (BUN), urine albumin creatinine ratio (uACR), mesangial matrix expansion (MME) and renal fibrosis compared to vehicle alone, without influencing glycemia or survival. However, H-2K^b^-ADSCs recipients had greater reductions in BUN and uACR, reduced intra-renal myeloid cell infiltration, increased splenic regulatory T cell (Treg) proportions and increased intra-renal Treg infiltration and FOXP3 and IL-10 mRNA. Nonetheless, recipients of H-2K^b^-ADSCs also had decreased splenic CD4/CD8 T cell ratios, increased circulating anti-H-2K^b^ IgG antibodies and histological and biochemical evidence of inflammatory liver injury. These novel findings demonstrated that ADSCs expressing an MHC-I allo-antigen had superior beneficial effects on DN than fully autologous ADSCs. Improved DN severity was associated with immune modulation, including Treg enhancement, but also had potentially detrimental immunological effects in mice with established diabetes. The results highlight the need for further investigation of the immune modulatory effects of Allo-MSCs in diabetes and its organ-specific complications.

## 1. Introduction

In 2021, the IDF Diabetes Atlas Tenth Edition reported that diabetes mellitus (diabetes) affected approximately 6.8% of the world population and projected prevalences of 7.6% by 2030 and 8.4% by 2045 [1]. Diabetic nephropathy (DN), now more commonly referred to in clinical medicine as diabetic kidney disease (DKD), is the most common microvascular complication of diabetes and is the main cause of mortality and disability in people with diabetes. The prevalence of DKD can reach 30% to 40% after 20 years of diabetes. Of these, 5 to 10% will progress to end-stage kidney disease [2,3,4]. Diabetic nephropathy is not caused by a single factor, but rather by various soluble mediators, cellular interactions and activation of different signaling pathways. In diabetes, periods of poor glycemic control along with other risk factors, such as hypertension and dyslipidemia, promote adverse cellular events within the kidneys, including increases in mitochondrial reactive oxygen species production, epigenetic changes and senescence, which contribute to a state of persistent activation of pro-fibrotic inflammatory pathways [5]. These diverse mechanisms of chronic inflammation make it difficult to target a single molecular mediator or pathway to reverse the progression of DN. Although, there is currently no treatment to fully halt the progression of DN, cellular therapies, most notably MSCs, have shown therapeutic promise because they offer the potential to simultaneously act on multiple disease mechanisms to actively promote tissue repair and regeneration [6,7]. Among the specific therapeutic effects that have been experimentally demonstrated for MSCs are the promotion of angiogenesis through paracrine factors and exosomes, inhibition of chronic inflammation [8], regulation of apoptosis and extracellular matrix dynamics, and promotion of the regeneration of damaged tissues [9,10]. The distinct ability of MSCs to modulate the immune system is one of their most compelling potential mechanisms of action in the treatment of DN [8]. Repeated and systemic administration of human adipose-derived MSCs (ADSCs) has also been shown to significantly reduce glomerular hypertrophy, tubular interstitial damage, the expression of the podocyte-specific proteins WT-1 and synaptopodin in animals with overt DN [9,11,12]. Recently, an early-phase clinical trial evaluated the safety and preliminary efficacy of a novel bone marrow-derived anti-CD362-selected allogeneic (Allo)-MSCs product (ORBCEL-M) in adult patients with T2DM and progressive DKD. The results showed a reduction in the rate of decline in estimated GFR in the ORBCEL-M group compared to a placebo group, as well as evidence of anti-inflammatory modifications to the circulating immune cell repertoire [13]. Thus, based on pre-clinical and clinical studies, MSCs appear to alleviate DN progression through a range of potential mechanisms, including modulation of pathogenic inflammation [14]. Autologous MSCs (Auto-MSCs) represent a safe therapeutic option for patients with DKD. However, in individuals with diabetes, Auto-MSCs may exhibit reduced proliferation, clonogenicity and differentiation capacity, and/or induced premature senescence and apoptosis [15]. Conversely, healthy donor Allo-MSCs may have greater anti-apoptotic, tissue repair and differentiation abilities than Auto-MSCs, but could also induce an immune response in recipients through the delivery of allo-antigens—in particular mismatched MHC proteins [16]. Therefore, repeated Allo-MSCs administrations within a short period of time have the potential to induce a strong immune response. Nonetheless, in clinical trials to date of Allo-MSCs, there has been minimal evidence of induction of immune responses against allogeneic human MHC (HLA) proteins [13,16]. Furthermore, experimental studies indicate that Allo-MSCs administration may induce immune responses against allogeneic MHC proteins that are skewed toward alternative activation pathways—possibly, as a result of the inherent immunosuppressive effects of MSCs on antigen presenting cells [17]. Thus, it remains unclear whether host immune responses to MSCs-delivered allo-antigens exert potentially adverse or beneficial effects when administered in the setting of chronic inflammatory conditions such as DKD. To address this issue, in the current study, we used a lentiviral transduction strategy to express an allogeneic mouse MHC class I protein (H-2K^b^) on the surface of ADSCs from DBA/2J (H-2K^d^) mice and determined their effects to mitigate features of DN in diabetic DBA/2J following single or repeated intravenous administrations in comparison to diabetic mice that received vehicle alone or empty-vector-transduced ADSCs (EV-ADSCs). Male DBA/2J mice, rendered diabetic by streptozotocin (STZ) injection, were selected for the study based on their well-described propensity to develop more overt clinical and pathological features of DN compared to other commonly used mouse strains [18,19].

## 2. Methods

### 2.1. Experimental Animals

Ethical approval for the project was received from the Institutional Animal Care and Use Committee of Hebei Medical University. Male DBA/2J mice were purchased at 4 weeks’ age from SPF Biotechnology Co., Ltd. (Beijing, China) and were housed in a Stratospheric feeding facility under 12 h of alternating light and dark feeding conditions until used for cell and tissue collection or for generation of a diabetes model. In total, 49 mice underwent interventional procedures across two separate experiments.

### 2.2. Isolation, Culture and Characterization of DBA/2J Mouse Adipose-Derived Mesenchymal Stem Cells

**Figure 1:**
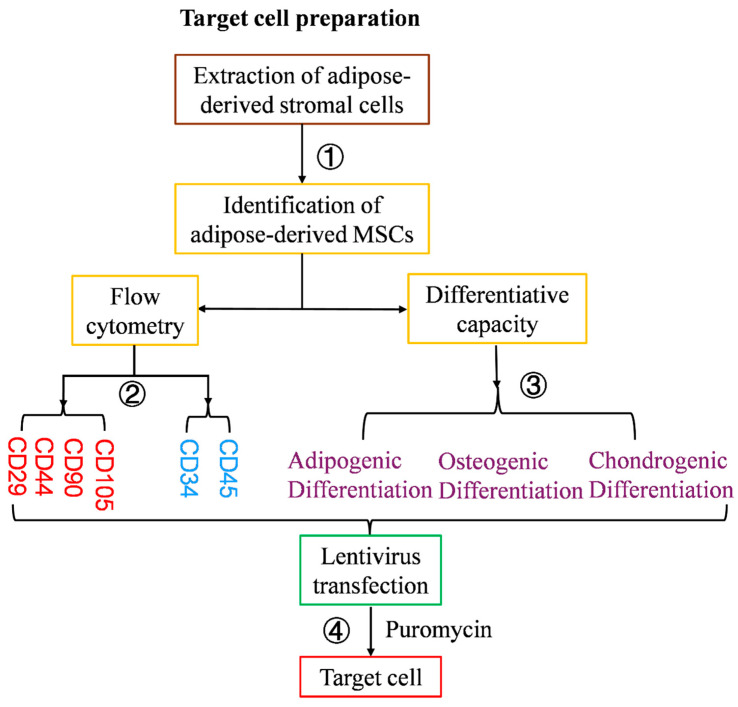
Summary of the sequence of steps for preparation, characterization and lentiviral transduction of DBA2/2J mouse adipose-derived mesenchymal stromal cells. ① Extraction and culture of adipose-derived mesenchymal stromal cells from the inguinal and epididymis of DBA/2J mice were extracted for culture. ② Flow cytometry of culture expanded ADSCs to confirm positive (red) and negative (blue) expression of relevant surface markers. ③ Confirmation of multi-lineage ADSC differentiation capacity. ④ Lentiviral transduction followed by puromycin selection.

The sequence of steps for ADSC isolation, culture expansion and characterization prior to lentiviral transduction is summarized in Figure 1. Male DBA/2J mice were euthanized, and inguinal and peri-epididymal white adipose tissue were aseptically dissected bilaterally, carefully removing visible blood vessels and connective tissue. The collected adipose tissue was washed in phosphate-buffered saline (PBS) containing penicillin and streptomycin to remove residual blood and debris, then minced into small fragments. The adipose tissue was then digested in 2% collagenase I for 45 min in a 37 °C water bath for 1 h, with gentle stirring every 5 min. Following this, the cell suspension was centrifuged at 1000 rpm for 5 min, and the pellet was re-suspended in PBS and digested in 2% collagenase at 37 °C for a further 15 min to optimize cell yield from incompletely dissociated tissue fragments. Next, the adipose tissue samples were centrifuged to obtain a cell pellet which was re-suspended and washed 3 times in PBS by centrifugation. After the final wash, the supernatant was discarded, 2 mL of complete culture medium (15%Fetal Bovine Serum + 85% Gibco Basic DMEM/F-12 + 1% penicillin and streptomycin) was added, and the cells were re-suspended and transferred to a 6 cm dish. An additional 3 mL of culture medium was added to the flask which was mixed gently and placed in a 37 °C, 5% CO_2_ incubator for culture (Figure 1①).

Once ADSC cultures had been passaged to the P3 generation, the cells were lifted with 0.25% trypsin, centrifuged at 1000 rpm for 5 min, re-suspended in 1 mL PBS, then counted and aliquoted into FACS tubes at concentrations of at least 2 × 10^5^ cells per tube. Six FACS tubes were prepared: a negative (unstained) control tube and tubes stained with the following fluorochrome-conjugated antibodies against the following mouse antigens for 1 h in the dark: CD29-PE, CD44-APC, CD105-PE, CD45-FITC, CD34-FITC. After staining, the cells were washed in 400 μL PBS and re-suspended and analyzed immediately by flow cytometry (BD bioscience) (Figure 1②).

To assess the trilineage differentiation potential of DBA/2J ADSCs, cells at P3 were cultured separately in osteogenic, chondrogenic, and adipogenic induction media according to standard protocols. The differentiation assays were performed as previously described using commercial kits according to the OriCell, China manufacturers’ protocols [20,21] (Figure 1③). Briefly, for adipogenesis, cells were cultured in an induction medium for 10–14 days, and differentiation was verified by Oil Red O staining, with the appearance of abundant intracellular lipid droplets indicating adipogenic commitment. Osteogenic differentiation was induced for 14–21 days and assessed using Alizarin Red staining to detect calcium mineral deposition, with distinct Alizarin Red-positive mineralized nodules confirming osteogenesis. Chondrogenic differentiation was performed using a pellet culture system in chondrogenic induction medium for 21 days, and evaluated by Alcian Blue staining, with intense blue sulfated glycosaminoglycan-rich matrix deposition indicating chondrogenic differentiation.

### 2.3. Lentiviral Transduction and Positive Selection of DBA/2J Mouse ADSCs

**Figure 2:**
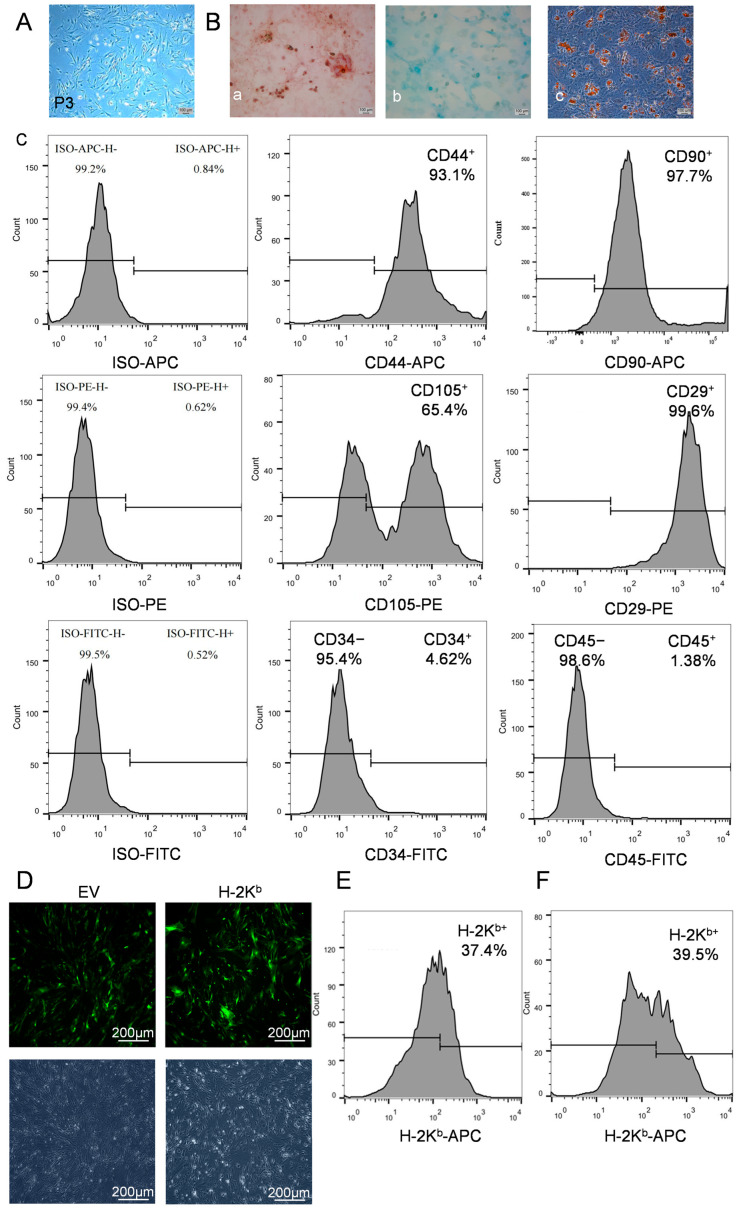
Preparation, characterization and lentiviral transduction of adipose-derived mesenchymal stromal cells from DBA/2J mice: Adipose mesenchymal stem cells were extracted from DBA/2J mice and cultured for 3 passages. (**A**). Representative phase contract light microscopic image of passage (P)3 DBA/2J ADSCs demonstrating spindle shape and spiral growth pattern. (**B**). Representative photomicrographs of DBA/2J ADSCs cultured in three induction differentiation media and demonstrating positive staining with Alizarin Red for osteogenesis (**a**), Alcian Blue for chondrogenesis (**b**), and Oil Red O for adipogenesis (**c**). (**C**). Flow cytometry histograms demonstrating surface staining and % positivity compared to isotype control staining of P3 DBA/2J ADSCs for CD44 (93.1%), CD90 (97.7%), CD105 (65.4%), CD29 (99.6%), CD34 (4.6%) and CD45 (1.4%). (**D**). Representative 100× images of fluorescence (upper) and corresponding bright field microscopy of DBA/2J ADSCs following empty vector (EV) and H-2K^b^-containing vector transduction using a GFP-expressing lentiviral vector at 100MOI and 50MOI, respectively, with puromycin selection. (**E**,**F**). Flow cytometry histogram demonstrating positive surface staining with anti-H-2K^b^ antibody of (**E**) unmodified ADSCs derived from H-2K^b^ genotype (C57BL/6) mice (37.4% positive) and (**F**) DBA/2J ADSCs transduced with a H-2K^b^-expressing lentiviral vector at 50MOI (39.5% positive).

A commercial lentiviral vector (Gv492) containing GFP reporter and puromycin resistance genes was obtained from Shanghai Genechem Co., Ltd., Shanghai, China and the cDNA sequence of the H-2K^b^ gene (transcript NM_001001892) was ligated into the vector. Preparations of the H-2K^b^-containing vector and the control (Empty) vector were generated and transferred into P3 ADSCs derived from DBA/2J mice using the vendor-recommended protocol. The recombinant lentivirus titers used were 1.00 × 10^8^ TU for H-2K^b^ lentivirus, and 5.00 × 10^8^ TU for the empty vector (EV). The number of cells plated for transduction was 5 × 10^4^ cells per well. The viruses were re-suspended in DMEM/F12 medium without bovine serum, and the total final volume of each transduction well was 1 mL. The transduction conditions were as follows: H-2K^b^ transduction: MOI = 50 (DMEM/F12: 935 μL, Virus 25 μL, infection enhancer A: 40 μL). EV transduction: MOI = 100 (DMEM/F12: 950 μL, virus 10 μL, infection enhancer A: 40 μL). The MOI for H-2K^b^ transduction was selected in preliminary experiments to result in an expression level of H-2K^b^ in DBA2/J ADSCs that was comparable to the constitutive expression level of H-2K^b^ in ADSCs derived from C57BL/6 mice. The MOI for EV transduction was selected based on GFP expression. After addition of the transduction reagents, the cells were gently mixed and cultured at 37 °C, 5% CO_2_ for 12 h, following which the culture medium was exchanged. After 24 h, the cells were passaged and puromycin was added to 4 μg/mL (Biosharp, Hefei, China, Catalog: 23188809). After 60 h, cells were observed for green fluorescence under a fluorescence microscope with DAPI staining of nuclei and %GFP^+^ was calculated (Figure 1④). Finally, transduction efficiency for the transgene was determined by flow cytometry using anti-H-2K^b^-APC antibody (Figure 2E,F).

### 2.4. Development of a Mouse Model of Diabetes

**Figure 3:**
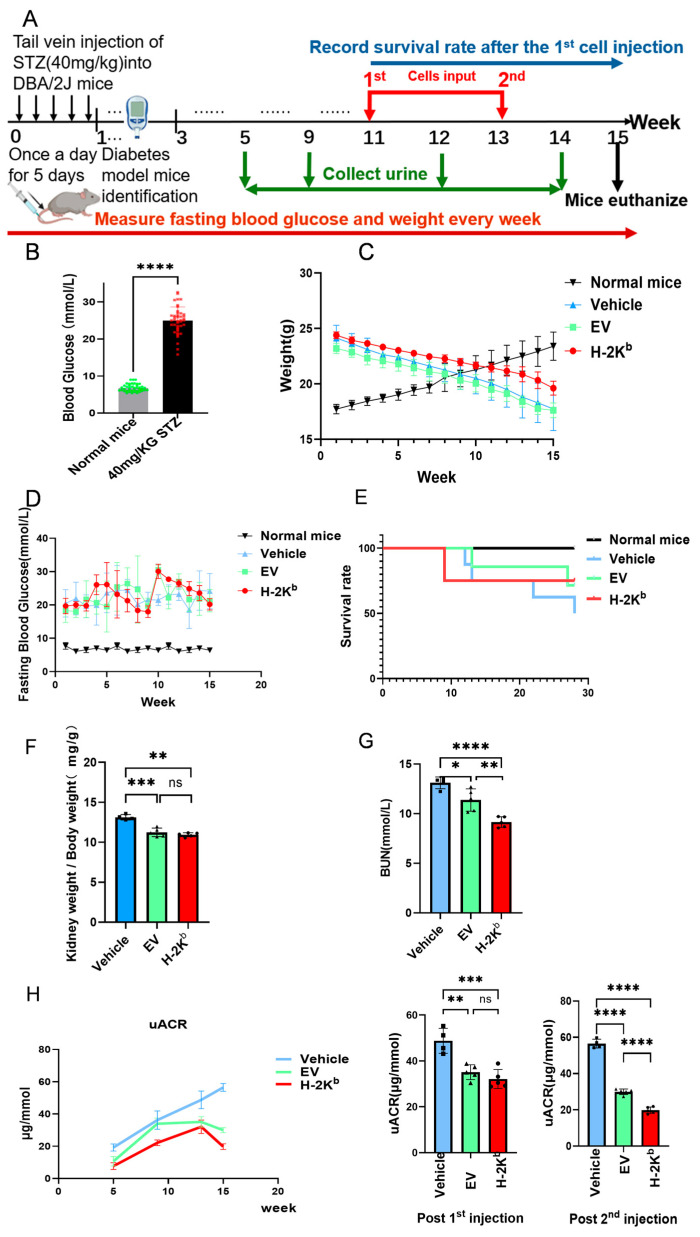
Physiological effects of two intravenous injections of H-2K^b^-expressing autologous ADSCs compared to empty vector-transduced ADSCs or vehicle only in a DBA/2J mouse model of diabetes and diabetic nephropathy: (**A**). Illustration summarizing the details and timeline of the in vivo experiment. (**B**). Graph depicting the results of blood glucose measurements of untreated and streptozotocin (STZ)-treated DBA/2J mice one week after completion of a sequence of 5 STZ injections. (**C**). Graph depicting serial measurement of body weight over the course of 15 weeks in a group of non-diabetic mice and three groups of diabetic mice that received two injections of either vehicle, empty-vector transduced ADCSs (EV) or H-2K^b^-expressing ADSCs (H-2K^b^). (**D**). Graph of serial blood glucose measurements over the course of 15 weeks from the same 4 groups of mice. (**E**). Kaplan–Meier survival curves from the day of first injection (0) to the day of euthanasia (28) of the same 4 groups of mice. (**F**). Graph depicting the results of kidney/total body weight ratios calculated at the time of euthanasia (week 15) for three groups of that received injections of either vehicle, empty-vector transduced ADCSs (EV) or H-2K^b^-expressing ADSCs (H-2K^b^). (**G**). Graph depicting the results of blood urea nitrogen (BUN) assays of the same three groups of diabetic mice. (**H**). Graphs depicting the results of calculated urine albumin creatinine ratio (uACR) from the same three groups of diabetic mice at 4 time-points during the 15-week experiment (left line graphs) and time-points after the first injection (post-1st injection) and shortly before euthanasia (post-2nd injection) (middle and right bar graphs). For column graphs, column heights represent group means, error bars represent SEM, and black dots represent results for individual samples. Statistical significance is indicated by * = *p* < 0.05, ** = *p* < 0.01, *** = *p* < 0.001, **** = *p* < 0.0001, ns = non-significant.

When male DBA/2J mice had reached 8 weeks’ age, diabetes was induced by daily intraperitoneal injection of STZ (Sigma Aldrich) at a concentration of 40 mg/kg in Saline for 5 days. One week after the first STZ injection, fasting blood glucose was measured, and a blood glucose value ≥11.1 mmol/L was considered to confirm diabetes. During the remaining 14 weeks of the experiment, the mice were monitored for fasting blood glucose every 2 weeks and body weight changes were measured weekly. At 11 weeks after the first injection of STZ, mice which had reached the blood glucose threshold for diabetes were randomized into three groups of *n* = 8 mice each and selected doses of H-2K^b^-ADSCs, EV-ADSCs and vehicle alone were administered via the tail vein. Group sizes were determined based on literature review with urine albumin creatinine ratio (uACR) as the primary outcome measure. Both H-2K^b^-ADSCs and EV-ADSCs were administered as 1 × 10^6^ cells/animal suspended in 100 μL of sterile saline while vehicle injections consisted of 100 μL of sterile saline. The cell dose was selected based on our previously published work [22] to be within the typically reported safe intravenous dose range of 0.5 to 2.0 × 10^6^ cells for adult mouse models of kidney disease [23]. At 13 weeks, the tail vein injections were repeated. Two weeks after the second injections (15 weeks after the first injection of STZ), all surviving animals in each group were euthanized by cervical dislocation under anesthesia. Regarding animal numbers, the initial group sizes for the three experimental groups (vehicle, EV-ADCSs and H-2K^b^-ADSCs) were eight mice each. In keeping with the relatively severe diabetic phenotype reported by others for the DBA2/J strain [18,19], attrition occurred among the animals in each group resulting in the following final group sizes: vehicle, *n* = 4 EV-ADSCs, *n* = 5, H-2K^b^-ADSCs, *n* = 5 (see Figure 3E). Furthermore, limitations in the health of individual mice at the time of euthanasia impacted the quality and/or sizes of the biological samples for some of the surviving animals, resulting in final numbers of *n* = 3–5/group for analyses carried at the terminal time-point of the study (as indicated by individual data-points in all graphs). For some experiments, samples from a group of five non-diabetic male DBA/2J mice housed in the same facility were collected to serve as normal controls.

Following completion of the primary experiment, a secondary experiment was performed by the same protocol and under identical conditions for the specific purpose of performing additional kidney tissue analyses of immune cell infiltration, cytokine mRNA levels and collagen deposition. This experiment included a group of non-diabetic DBA/2J mice (*n* = 6) and groups of diabetic DBA/2J mice treated with vehicle (*n* = 4), EV-ADSCs (*n* = 5), and H-2K^b^-ADSCs (*n* = 5).

Regarding blinding procedures, group assignment of animals was generated and recorded by an independent researcher who was not involved in any experimental procedures. One team member responsible for administering treatments was aware of group allocation to ensure correct intervention, while all other personnel involved in routine handling, monitoring and outcome assessments remained blinded. For analyses of biological samples from mice, measurements were blinded to group allocation. All samples and images were labeled with coded identifiers until assessment was completed, at which time unblinding was performed.

### 2.5. Collection and Analysis of Biological Samples from Mice

Blood glucose testing was performed using a standard clinical glucometer on small volume tail-prick blood samples obtained using a sterile lancet. Glucose monitoring was performed in the afternoon with the mice fasting for 6 h beforehand. For serum collection at the end of the experiment, blood was collected under terminal anesthesia from the retro-orbital sinus via the medial canthus. Approximately 400 µL to 1 mL of whole blood was collected per mouse using 0.5 × 100 mm capillary tubes. Blood samples were transferred into 1.5 mL microcentrifuge tubes, placed in room temperature for 2 h, then centrifuged at 3000 rpm, 4 °C for 20 min. The serum was then carefully transferred to cryotubes and stored at −80 °C. Approximately 1 mL of urine was collected from each mouse by timed placement in metabolic cages. The collected urine was centrifuged at 3000 rpm, 4 °C for 20 min, following which the supernatants were transferred to cryotubes and stored at −80 °C. For tissue collections, kidneys, pancreas, and liver were dissected from each animal following euthanasia. Portions of each organ were fixed in formalin and were subsequently paraffin embedded or stored as frozen tissue at −80 °C. Additional tissue portions were snap frozen and stored at −80 °C for subsequent mRNA extraction. Spleens were dissected and used fresh for multi-parameter flow cytometry analysis.

### 2.6. Histological Analysis of Kidney and Liver Tissue Sections

Four-micron thick sections were cut from formalin-fixed, paraffin-embedded blocks of kidney and liver tissues onto glass slides using a microtome and were subsequently used for histological staining. Hematoxylin and Eosin (H&E) staining: Kidney and liver tissue sections on glass slides were de-waxed, hydrated and stained using the Solarbio G1120 kits (Beijing Solarbio Science, Beijing, China) according to the manufacturer’s instructions. Masson trichrome staining: Kidney tissue sections on glass slides were de-waxed, hydrated and stained using the Solarbio G1340 kit according to the manufacturer’s instructions. Periodic Acid Schiff (PAS) staining: Kidney tissue sections on glass slides were de-waxed, hydrated and stained using the Solarbio G1281 kit according to the manufacturer’s instructions. Modified Sirus Red Stain Kit (No Picric Acid): Kidney tissue sections on glass slides were de-waxed, hydrated and stained using the Solarbio G1472 kit according to the manufacturer’s instructions.

Numbers of infiltrating inflammatory cells in the peri-glomerular regions were counted in blinded fashion in 200× images of individual glomeruli from representative H&E-stained kidney tissue sections from each experimental group and from non-diabetic male DBA/2J mice. Infiltrating cells, likely to represent mixtures of inflammatory cell types including monocyte/macrophages and lymphocytes, were identified as cells with smaller, darkly stained nuclei within the peri-glomerular interstitium. Glomerular size (area) was quantified from the same representative images of individual H&E-stained glomeruli using ImageJ 1.54g software. Mesangial area and glomerular/peri-glomerular fibrosis were measured from 400× digital images of individual glomeruli from PAS- and Masson’s trichrome-stained kidney tissue sections, respectively, using ImageJ analysis software. For mesangial area, images were converted to greyscale, each glomerulus was isolated as the region of interest (ROI), the density of the mesangial region was defined as the analysis parameter and the proportion of the ROI containing this density was automatically calculated. For quantification of glomerular fibrosis on Masson trichrome-stained sections, the total image (glomerulus and peri-glomerular region) was defined as the ROI, the deep blue color density of collagen-containing tissue was defined as the analysis parameter and the proportion of the ROI containing this color was automatically calculated. For both analyses, %ROIs were quantified for 6–10 individual full-profile glomeruli randomly selected from 3–5 separate cortical regions per animal. The final result for each group represented the mean ± SEM of the average %ROIs of all animals in the group. For quantification of collagen in Sirus red-stained sections, kidney tissue sections were initially examined at a 40× magnification to identify areas of red staining. Subsequently, high-resolution images were captured at 200× magnification. Three randomly selected regions of interest were quantitatively analyzed using ImageJ software to determine the proportion of red-stained collagen fibers within the microscopic field. The mean value derived from these measurements was recorded as the final value for each kidney. The final result for each group represented the mean ± SEM of the average values of all animals in the group.

For H&E-stained liver sections, hepatocellular damage, including necrosis, was qualitatively assessed based on established histomorphological criteria (hepatocyte ballooning, cytoplasmic eosinophilia, nuclear condensation or loss, and disruption of hepatic architecture) along inflammatory cell infiltration. Histological assessments were independently evaluated by a pathology specialist who was blinded to group allocation.

### 2.7. Enzyme-Linked Immunosorbent Assays (ELISA) to Detect Urine Albumin and Creatinine, Blood Urea Nitrogen and Serum ALT, AST and Total Bilirubin

Urine albumin (ALB) and creatinine (UCR) concentrations, as well as blood urea nitrogen (BUN), alanine aminotransferase (ALT), aspartate aminotransferase (AST), and total bilirubin (TBIL) concentrations in serum were measured using commercially available ELISA kits from Shanghai Zhuocai Biotechnology Co., Ltd. (Shanghai, China) according to the manufacturers’ instructions. The optical density was measured at 450 nm using a microplate reader, and concentrations were calculated based on standard curves.

### 2.8. Serum Anti-H-2K^b^-gG Detection

Freshly prepared erythrocyte-free C57BL/6 (H-2K^b+^) mouse spleen cells were suspended in PBS at 8 × 10^6^ cells/mL. Purified anti-H-2K^b^ (positive control) (eBioscience, San Diego, CA, USA, anti-mouse H-2K^b^) and serum samples were added and incubated for 30 min. The cells were then washed and re-suspended in PBS and incubated for 30 min with aliquots of goat anti-mouse IgGFc F(ab)-FITC at a dilution of 1:400 to detect bound IgG antibody and with anti-mouse T cell receptor (TCR)-β-PE to distinguish T cells from non-T cells. Finally, the cells were washed twice and re-suspended in PBS and were analyzed immediately by flow cytometry.

### 2.9. Reverse Transcription and Quantitative Polymerase Chain Reaction (RT-qPCR)

Frozen kidney tissue was ground using the Trizol method and a grinder, and the total RNA in the tissue was extracted with chloroform, precipitated with isopropanol and absolute ethanol, and the RNA concentration and quality were measured by BioTek (Winooski, VT, USA), Synergy HT. Removal of genomic DNA and reverse transcription to obtain single strand cDNA was performed using RNA Reversal Instrument (Bio-Rad Laboratories, Inc., Hercules, CA, USA). PCR reactions and CT value determinations were then performed in a 10 μL PCR reaction system using the Quantifast SYBR Green PCR Master Mix method and the 7500 Realtime PCR instrument (ABI, Waltham, MA, USA). The ΔCT values were obtained with a house-keeping gene β-actin.

The following Primer sequences were custom-designed and synthesized by Sangon Biotech (Shanghai) Co., Ltd. (Shanghai, China):
β-actin-FORWARD-Mouse5′-AGAGGGAAATCGTGCGTGACA-3β-actin-REVERSE-Mouse5′-CACTGTGTTGGCATAGAGGTC-3′IL-6-FORWARD-Mouse5′-TACCACTTCACAAGTCGGA-3′IL-6-REVERSE-Mouse5-AATTGCCATTGCACAACTC-3TNF-α-FORWARD-Mouse5′-CACCACCATCAAGGACTCAA-3′TNF-α-REVERSE-Mouse5′-GAGACAGAGGCAACCTGACC-3′TGF-β-FORWARD-Mouse5′-ACCAAGGAGACGGAATACAG-3′TGF-β-REVERSE-Mouse5′-CGTTGATTTCCACGTGGAG-3Foxp3-FORWARD-Mouse5′-TTACTCGCATGTTCGCCTACTTCAG-3′Foxp3-REVERSE-Mouse5′-CTCGCTCTCCACTCGCACAAAG-3′

### 2.10. Terminal Deoxynucleotidyl Transferase dUTP Nick-End Labeling (TUNEL) Immunofluorescence Staining of Liver Tissue Sections

TUNEL immunofluorescence staining of liver tissue sections was performed by SercviceBio, Co., Ltd., Wuhan, China. Briefly, sections of paraffin-embedded liver tissue were dewaxed in xylene and ethanol, washed in water, and incubated in proteinase K solution followed by membrane permeabilization. The sections were then incubated with TUNEL reaction solution followed by DAPI staining solution and were sealed with anti-fluorescein quencher. Photomicrographs of the stained slides were generated using immunofluorescence microscopy and the % area with positive fluorescein staining was quantified using image analysis software.

### 2.11. Immunofluorescence Staining of Kidney Sections

Immunofluorescence staining was performed to detect and quantify CD11b-, CD45- and FOXP3-expressing cells in kidney tissue sections. Paraffin-embedded sections were deparaffinized and rehydrated, followed by antigen retrieval, hydrogen peroxide blocking, and serum blocking according to standard protocols. For FOXP3 staining, sections were incubated in 0.25% Triton X-100 for 20 min at room temperature to permeabilize cells. Sections were then incubated overnight at 4 °C with rabbit anti-mouse CD11b primary antibody (1:750 dilution; Hangzhou Huaan Biotechnology, Hangzhou, China), anti-mouse CD45 (1:500; Proteintech, Wuhan, China), or rabbit anti-FOXP3 (1:800; Abiowell, Nanjing, China). After three 5 min washes in PBST, sections were incubated with AF488-coupled goat anti-rabbit IgG (H+L), antibody for CD11b and FOXP3 (1:200 dilution; Ruipate Bio & Technology, Shijiazhuang, China) or TRITC-coupled goat anti-mouse IgG (H+L) antibody for CD45 (1:200 dilution; Ruipate Bio & Technology, China), for 1 h at room temperature. Following three 5 min washes in PBST, sections were counterstained with DAPI, treated to quench tissue autofluorescence, and mounted for imaging. Fluorescence signals for FITC, TRITC and DAPI were captured using an Olympus BX63 (Olympus Corporation, Tokyo, Japan) fluorescence microscope under identical acquisition settings. Quantification of fluorescence was performed on images (*n* ≥ 4 mice in each group with 3–5 non-overlapping cortical fields per tissue sample) analyzed at identical magnification. Quantification of CD11b, CD45, and FOXP3 positivity was performed by ImageJ 1.54g. The mean values for each group were used for statistical analysis using pre-defined tests. All staining and imaging procedures were repeated in at least two independent experiments. For each mouse, image acquisition and quantitative analysis were performed in a blinded manner with respect to group allocation.

### 2.12. Statistical Analysis and Analysis Software

All data were analyzed by GraphPad Prism v9 software. Inter-group statistical differences were determined by unpaired Student’s *t*-tests based on the absence of differences in *p* values in the F-test for homogeneity of variance analysis. Statistical significance was assigned to *p* values of <0.05. Standard curves for ELISA results were fitted by MasterPlex software (version 2.0.0.76). Flow cytometry data were analyzed by FlowJo v10.0 software. Stained tissue sections were analyzed by ImageJ-Pro Plus V6.0 software.

## 3. Results

### 3.1. Lentiviral Transduction Resulted in Successful Expression of H-2K^b^ on H-2K^d^ Genotype-Derived ADSCs

Adipose-derived MSCs were generated from DBA/2J (H-2K^d^) mice and were cultured to P3 generation (Figure 2A). Their osteogenic, chondrogenic and adipogenic potential was confirmed by differentiation assays (Figure 2B) and their expected surface marker phenotype (CD29^+^, CD44^+^, CD105^+^, CD90^+^, CD45^−^, CD34^−^) was validated by flow cytometry (Figure 2C).

The recombinant lentivirus with H-2K^b^ or the empty lentiviral vector (EV) were then transduced into DBA/2J ADSCs and puromycin-resistant ADSC colonies appeared after 60 h of selection. For both H-2K^b^ and EV transductions, fluorescence microscopy was used to count total (DAPI^+^) and transduced (GFP^+^) cells, with a positive rate of approximately 80% of the puromycin-selected cells displaying green fluorescence (Figure 2D). Following optimization of the protocol, flow cytometry using an H-2K^b^-specific monoclonal antibody confirmed surface expression of H-2K^b^ on the transduced DBA/2J ADSCs that was comparable to the natural expression by ADSCs derived from C57BL/6 (H-2K^b^ genotype) mice (Figure 2F compared to Figure 2E). Subsequently, the transduced DBA/2J ADSC preparations were referred to as H-2K^b^-ADSCs and EV-ADSCs.

### 3.2. Indicators of Diabetic Nephropathy Were Ameliorated to a Greater Extent by Repeated Intravenous Injection of H-2K^b^-ADSCs Compared to EV-ADSCs

The sequence and timeline of the in vivo experiment is summarized in Figure 3A. As shown, male DBA/2J mice were induced to develop diabetes by a 5-day STZ administration protocol. Two weeks later, blood glucose values varied between 29.9 mmol/L and 21.1 mmol/L indicating successful establishment of diabetes (Figure 3B). Subsequently, at 11 weeks and 13 weeks after initiation of STZ injections, 1 × 10^6^ H-2K^b^-ADSCs or EV-ADSCs or the equivalent volume of vehicle were administrated by tail vein to groups of diabetic DBA/2J mice, which were then observed for a further 2 weeks before humane euthanasia (Figure 3A). As shown in Figure 3C, all three groups of diabetic mice gradually lost weight throughout the observation period compared to gain of weight observed in a group of non-diabetic mice (with lower starting weight due to younger age). There was a non-significant trend toward slower rate of weight loss in the H-2K^b^-ADSCs group. Trends in fasting blood glucose throughout the experiment indicated persistent hyperglycemia of approximately 20 mmol/L or greater (Figure 3D). Survival of animals from the time of the first ADSCs/vehicle injection to euthanasia (a total of 28 days) was also no different among the three experimental groups (Figure 3E).

At the end of the experimental protocol (15 weeks), the renal hypertrophy index was calculated by the ratio of kidney weight to body weight ([Kidney weight (mg)/Body weight (g)]) for surviving animals in the three diabetic groups. As shown in Figure 3F, the index was lower for both EV-ADSC and H-2K^b^-ADSC groups compared to the vehicle group, indicating an ameliorating effect in both ADSC types. In contrast, while blood urea nitrogen (BUN), an indicator of renal function, was significantly lower at week 15 in the EV-ADSC compared to the vehicle group, it was further reduced in mice from the H-2K^b^-ADSC group (Figure 3G). Finally, urine creatinine and urine albumin concentrations were quantified in urine collected from mice in the three diabetic groups using metabolic cages at weeks 5, 10, 13 (following first administrations) and 15 (following second administrations) of the experimental protocol. From these, urine albumin creatinine ratios (ACR) were calculated. As shown in Figure 3H, these analyses demonstrated similar significant decreases in urine ACR in the ADSC-treated groups compared to the vehicle group after the first administration while, after the second administration, urine ACR was significantly lower in the H-2K^b^-ADSC group than in both vehicle and EV-ADSC groups.

Overall, it was concluded that repeated intravenous injections of both EV (Auto)-ADSCs and H-2K^b^ (Allo)-ADSCs ameliorated indices of DN severity without influencing glycemia. Notably, the effect sizes for ADSC administration compared to vehicle for key renal endpoints such as BUN and uACR were of clinically meaningful magnitude. Furthermore, the effects of H-2K^b^-ADSCs were more potent by the end of the experimental protocol.

### 3.3. Repeated Intravenous Administration of Both EV-ADSCs and H-2K^b^-ADSCs Alleviated Pathological Abnormalities of Diabetic Nephropathy

**Figure 4:**
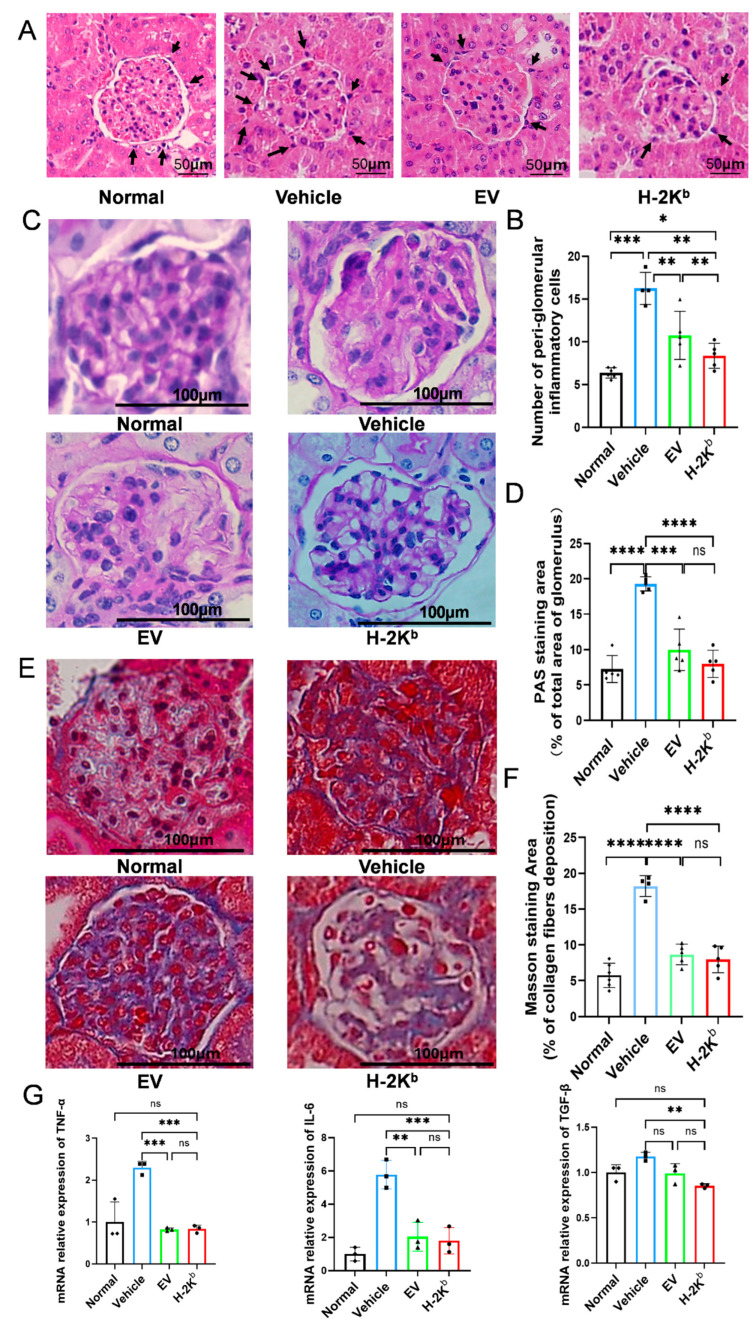
Renal effects of two intravenous injections of H-2K^b^-expressing autologous ADSCs compared to empty vector-transduced ADSCs or vehicle only in a DBA/2J mouse model of diabetic nephropathy: (**A**). Representative 200× photomicrographs of hematoxylin and eosin (H&E) stained sections of kidney tissue samples from non-diabetic DBA/2J mice (normal) and from diabetic DBA/2J mice that received two injections of either vehicle, empty-vector transduced ADCSs (EV) or H-2K^b^-expressing ADSCs (H-2K^b^). Arrows indicate infiltrating cells. (**B**). Graphical depiction of the results of quantitative analysis of the number of peri-glomerular infiltrating cells in images of individual glomeruli from each of the groups. (**C**). Representative 400× photomicrographs of individual glomeruli from periodic acid Schiff (PAS)-stained sections of kidney tissue samples from the same 4 groups of DBA/2J mice. (**D**). Graphical depiction of the results of quantitative image analysis of mesangial area (% PAS positive staining of total glomerular area) in PAS-stained kidney tissue sections from groups of diabetic DBA/2J mice that received two injections of either vehicle, empty-vector transduced ADCSs (EV) or H-2K^b^-expressing ADSCs (H-2K^b^). (**E**). Representative 400× photomicrographs of individual glomeruli from Masson’s trichrome stained sections of kidney tissue samples from the same 4 groups of DBA/2J mice. (**F**). Graphical depiction of the results of quantitative image analysis of blue staining area (% positive of total glomerular and interstitial area) in Masson’s trichrome-stained kidney tissue sections from the same 4 groups of DBA2/J mice. (**G**). Graphs depicting the results of quantitative RT-PCR of kidney tissue-derived messenger (m)RNA from the same 4 groups of DBA/2J mice as for Panel A for the cytokines tumor necrosis factor alpha (left, TNF-α), interleukin 6 (middle, IL-6) and transforming growth factor beta (right, TGF-β). For column graphs, column heights represent group means, error bars represent SEM, and black dots represent results for individual samples. Statistical significance is indicated by ns = non-significant, * = *p* < 0.05, ** = *p* < 0.01, *** = *p* < 0.001, **** = *p* < 0.0001.

To screen for glomerular abnormalities, H&E staining was performed on kidney sections prepared from representative animals within each diabetic group at the end of the experimental protocol (Figure 4A). Glomerular structure was clearly abnormal in diabetic sections compared to non-diabetic, although glomerular volume (area) analysis did not demonstrate differences among the experimental groups. However, the quantification of peri-glomerular infiltrating cells indicated increased cell numbers in the vehicle group compared to non-diabetic animals and significantly lower cell numbers in the H-2K^b^-ADSC and EV-ADSC groups compared to the vehicle group, as well as significantly lower cell numbers in the H-2K^b^-ADSC group compared to the EV-ADSC group (Figure 4B). Subsequently, quantitative analysis of PAS-stained sections indicated that mesangial matrix expansion was significantly lower in kidneys of both ADSC-treated groups compared to kidneys of vehicle-treated mice with a trend toward lower mesangial area in the H-2K^b^-ADSCs group (Figure 4C,D). Masson trichrome staining was used to quantitatively analyze renal interstitial and glomerular/periglomerular fibrosis. In the vehicle group, substantial collagen deposition in glomerular mesangium and peri-glomerular renal interstitium was observed. In contrast, the levels of glomerular/periglomerular collagen deposition were significantly reduced in both the EV-ADSC and H-2K^b^-ADSC groups compared to vehicle and normal group (Figure 4E,F). These results indicated that repeated intravenous injections of both Auto- and Allo-ADSCs protected against the progression of specific pathological features of DN. Quantitative mRNA analysis of kidney tissue samples indicated that intrarenal expression of the cytokines TNF-α, IL-6 and TGF-β was significantly lower in both EV-ADSC and H-2K^b^-ADSC groups compared to kidneys of both vehicle and non-diabetic groups (Figure 4G).

**Figure 5:**
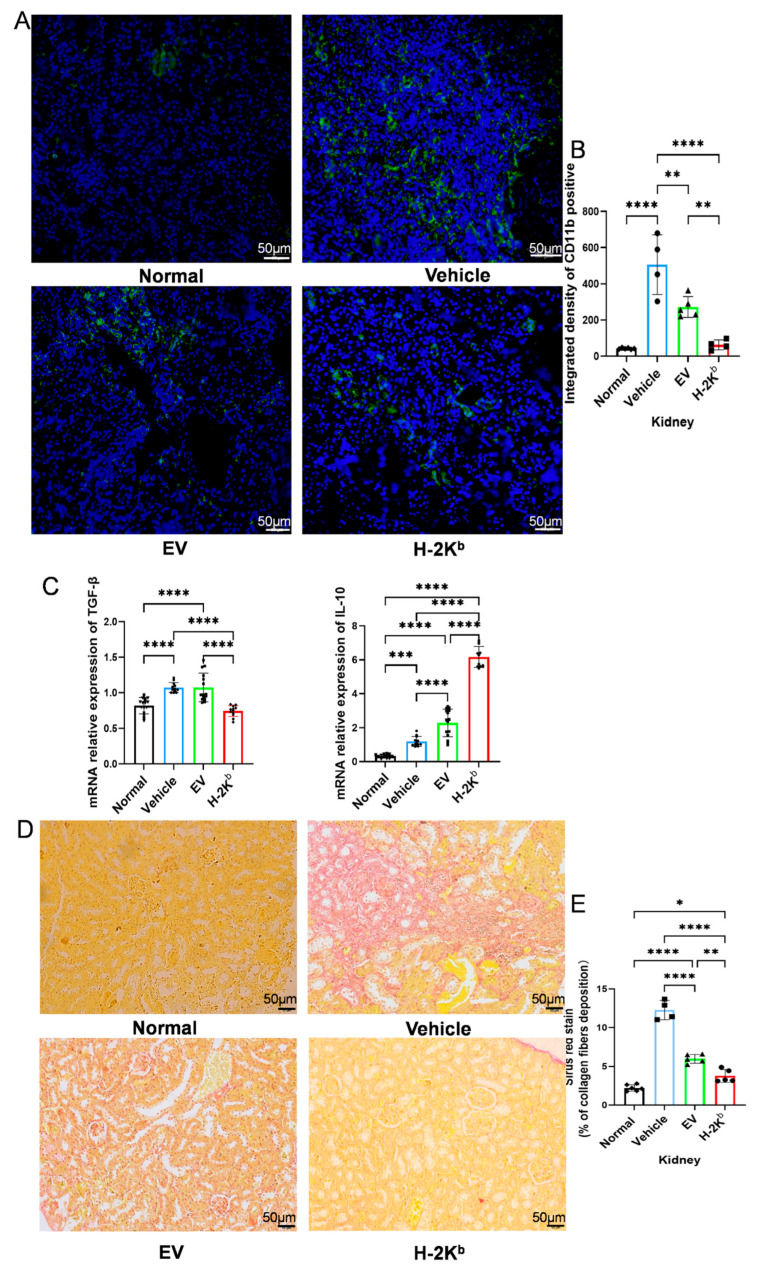
Effects of two intravenous injections of H-2K^b^-expressing autologous ADSCs compared to empty vector-transduced ADSCs or vehicle only on myeloid cell infiltration and fibrosis in a DBA/2J mouse model of diabetic nephropathy: (**A**). Representative 200× fluorescence photomicrographs of CD11b (green) and DAPI (blue) staining of kidney tissue sections from non-diabetic DBA/2J mice (normal) and from diabetic DBA/2J mice that received two injections of either vehicle, empty-vector transduced ADCSs (EV) or H-2K^b^-expressing ADSCs (H-2K^b^). (**B**). Graphical depiction of the results of quantitative analysis of integrated density of CD11b staining from each of the four groups. (**C**). Graphs depicting the results of quantitative RT-PCR of kidney tissue-derived messenger (m)RNA from the same 4 groups of DBA/2J mice for TGF-β (left) and interleukin 10 (right, IL-10). (**D**). Representative 200× photomicrographs of Sirus Red staining of kidney tissue sections from the same 4 groups of DBA/2J mice. (**E**). Graphical depiction of the results of quantitative analysis of collagen fiber deposition (red staining) from each of the four groups. For column graphs, column heights represent group means, error bars represent SEM, and black dots represent results for individual samples. Statistical significance is indicated by * = *p* < 0.05, ** = *p* < 0.01, *** = *p* < 0.001, **** = *p* < 0.0001.

**Figure 6:**
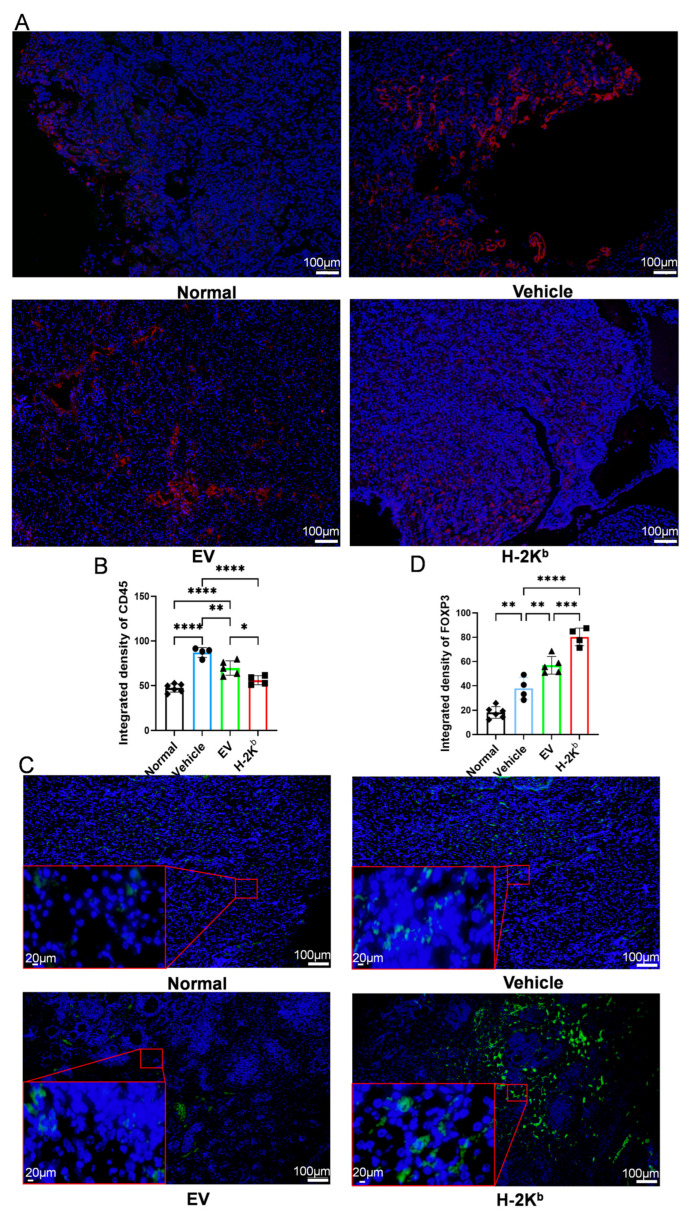
Effects of two intravenous injections of H-2Kb-expressing autologous ADSCs compared to empty vector-transduced ADSCs or vehicle only onCD45 and FOXP3 infiltration in a DBA/2J mouse model of diabetic nephropathy: (**A**). Representative 100× fluorescence photomicrographs of CD45 (red) and DAPI (blue) staining of kidney tissue sections from non-diabetic DBA/2J mice (normal) and from diabetic DBA/2J mice that received two injections of either vehicle, empty-vector transduced ADCSs (EV) or H-2K^b^-expressing ADSCs (H-2K^b^). (**B**). Graphical depiction of the results of quantitative analysis of integrated density of CD45 staining from each of the four groups. (**C**). Representative 100× and 400× fluorescence photomicrographs of FOXP3 (green) and DAPI (blue) staining of kidney tissue sections from non-diabetic DBA/2J mice (Normal) and from diabetic DBA/2J mice that received two injections of either vehicle, empty-vector transduced ADCSs (EV) or H-2K^b^-expressing ADSCs (H-2K^b^). (**D**). Graphical depiction of the results of quantitative analysis of integrated density of FOXP3 staining from each of the four groups. For column graphs, column heights represent group means, error bars represent SEM, and black dots represent results for individual samples. Statistical significance is indicated by * = *p* < 0.05, ** = *p* < 0.01, *** = *p* < 0.001, **** = *p* < 0.0001.

In order to further investigate the influence of EV- and H-2K^b^-ADSCs on intra-renal myeloid cell infiltration and interstitial collagen deposition, kidney tissue sections from a secondary experiment with similar groups of non-diabetic and diabetic DBA2/J mice were subjected to immunofluorescence staining for the pan-myeloid cell marker CD11b, Sirius Red staining to more accurately quantify collagen abundance, and RT-qPCR for TGF-β and IL-10 (Figure 5). As shown in Figure 5A,B, kidneys of vehicle-treated diabetic mice demonstrated increased staining density for CD11b compared to those of non-diabetic mice, indicating increased myeloid cell infiltration. Kidneys from both EV-ADSC- and H-2K^b^-ADSC groups had significantly lower CD11b staining compared to the vehicle group. Furthermore, CD11b staining was significantly lower in kidneys from the H-2K^b^-ADSC group compared to the EV-ADSC group. Similar patterns were observed for renal TGF-β mRNA expression (Figure 5C) and for Sirius Red staining of kidney tissue sections (Figure 5D). In contrast, renal mRNA expressions for the anti-inflammatory cytokine, IL-10, were increased in the EV-ADSC and H-2K^b^-ADSC groups compared to non-diabetic controls and to the vehicle-treated diabetic group. Furthermore, IL-10 mRNA was significantly higher in kidneys of H-2K^b^-ADSC- compared to EV-ADSC-treated animals (Figure 5C). Subsequently, kidney tissue sections from the same groups of animals were subjected to immunofluorescence staining for the pan-immune cell marker CD45 and the Treg-specific marker FOXP3 (Figure 6). As shown, CD45 staining was reduced (Figure 6A,B) and FOXP3 staining was increased (Figure 6C,D) in kidneys from the EV-ADSC and H-2K^b^-ADSC groups compared to the vehicle-treated diabetic group. Notably, renal FOXP3 fluorescence was also significantly higher in H-2K^b^-ADSC- compared to EV-ADSC-treated animals. These additional results strengthened the evidence that intravenous injections of both Auto (EV)-ADSCs and Allo (H-2K^b^)-ADSCs reduced intra-renal inflammation and fibrosis and increased Treg infiltration in DN, with the effect of Allo-ADSCs being more potent.

### 3.4. Repeated Administration of H-2K^b^-ADSCs Modulated Systemic T Cell and Antigen Presenting Cell Proportions and Promoted Treg in Diabetic Mice

**Figure 7:**
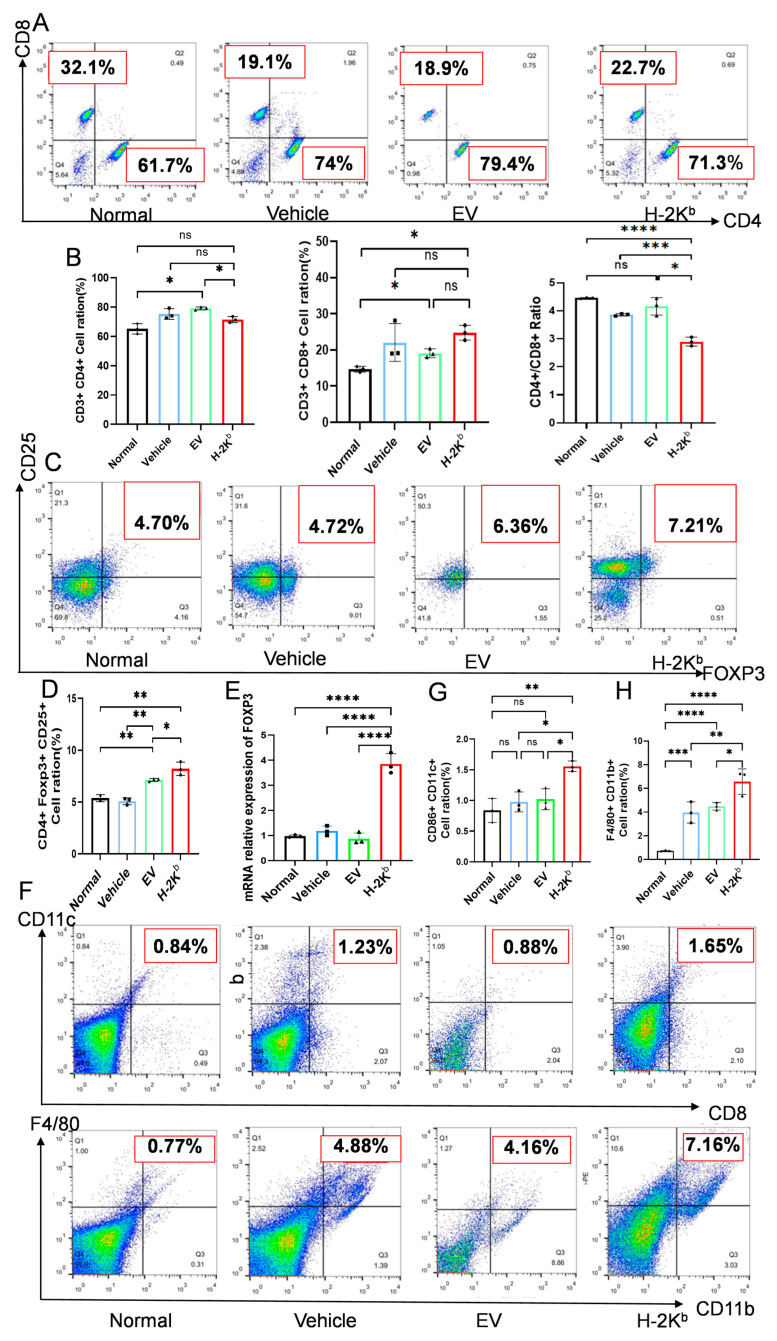
Immunological effects of two intravenous injections of H-2K^b^-expressing ADSCs compared to empty vector-transduced ADSCs or vehicle only in a DBA/2J mouse model of diabetes and diabetic nephropathy: (**A**). Representative flow cytometry dot plots demonstrating relative quantification of CD4^+^ and CD8^+^ T cells within splenocyte suspensions prepared from non-diabetic DBA/2J mice (normal) and from diabetic DBA/2J mice that received two injections of either vehicle, empty-vector transduced ADCSs (EV) or H-2K^b^-expressing ADSCs (H-2K^b^). Dot plots are gated on total CD3^+^ cells. (**B**). Graphs depicting the proportions of CD4^+^ T cells (left) and CD8^+^ T cells (middle) and the calculated CD4/CD8 T cell ratios in splenocyte suspensions prepared from the four groups of mice at the end of the experiment. (**C**). Representative flow cytometry dot plots demonstrating relative quantification of FOXP3^+^/CD25^+^ T cells among the total CD4^+^T cells within splenocyte suspensions prepared from the four groups of mice. (**D**). Graph depicting the proportions of FOXP3^+^/CD25^+^ T cells among the total CD4^+^ T cells within splenocyte suspensions from the four groups of mice. (**E**). Graph depicting the relative expression of mRNA for FOXP3 by qRT-PCR of kidney tissues samples from the four groups of mice. (**F**). Representative flow cytometry dot plots demonstrating relative quantification of CD86^+^/CD11c^+^ dendritic cells (upper) and F4/80^+^/CD11b^+^ macrophages (lower) among the total viable cells within splenocyte suspensions prepared from the four groups of mice. (**G**,**H**). Graphs depicting the proportions of CD86^+^/CD11c^+^ dendritic cells (**G**) and F4/80^+^/CD11b^+^ macrophages (**H**) in splenocyte suspensions prepared from the four groups of mice at the end of the experiment. For column graphs, column heights represent group means, error bars represent SEM, and black dots represent results for individual samples. Statistical significance is indicated by ns = non-significant, * = *p* < 0.05, ** = *p* < 0.01, *** = *p* < 0.001, **** = *p* < 0.0001.

To address potential immune modulatory effects of EV- and H-2K^b^-ADSCs in the setting of diabetes and DN, flow cytometric analyses were performed on splenocyte suspensions prepared from each of the diabetic groups at the end of the experimental protocol. Analysis of helper (CD4^+^) and cytotoxic (CD8^+^) T cell proportions among total T cells (CD3^+^) was carried out using the gating strategy shown in Figure 7A and was compared to samples from non-diabetic mice. The results indicated that CD4^+^ T cell proportions were lower and CD8^+^ T cell proportions were higher in spleens from the H-2K^b^-ADSC- group compared with the vehicle and EV-ADSC groups, resulting in a significantly lower splenic CD4/CD8 ratio in H-2K^b^-ADSCs-treated diabetic mice (Figure 7B). Furthermore, the proportions of splenic CD8^+^ T cells that expressed the activation marker CD25 was higher in the H-2K^b^-ADSCs group. However, flow cytometry analysis of CD4^+^/CD25^+^/FOXP3^+^ Treg also revealed that the H-2K^b^-ADSC group had a higher proportion of Treg among total splenic CD4^+^ T cells than all other groups (Figure 7C,D). Furthermore, quantitative analysis of FOXP3 mRNA levels in kidney tissue samples from the four groups revealed significantly higher intra-renal expression in the H-2K^b^-ADSCs group (Figure 7E). Finally, the proportions of CD11c^+^/CD86^+^ dendritic cells (DCs) and CD11b^+^/F4/80^+^ macrophages among total spleen cells were analyzed for the three diabetic groups. As shown in Figure 7F,G, both types of myeloid antigen presenting cells (APCs) were present at higher proportions in spleens of H-2K^b^-ADSCs-treated mice compared to those of vehicle and EV-ADSCs-treated mice.

From these analyses, we concluded that H-2K^b^-expressing (Allo)-ADSCs induced distinct modulations of the T cell and APC compartments in diabetic animals in comparison to vehicle- and EV (Auto)-ADSC-treated animals. These included increased proportions of activated CD8^+^ T cells as well as higher proportions of Treg within the CD4^+^ T cell compartment and proportionate increases in DCs and macrophages—suggesting potential for both immunogenic and immunosuppressive effects.

### 3.5. Repeated Administration of H-2K^b^-ADSCs Administration Resulted in Production of IgG Specific to H-2K^b^ and Liver Inflammation in Diabetic Mice

**Figure 8:**
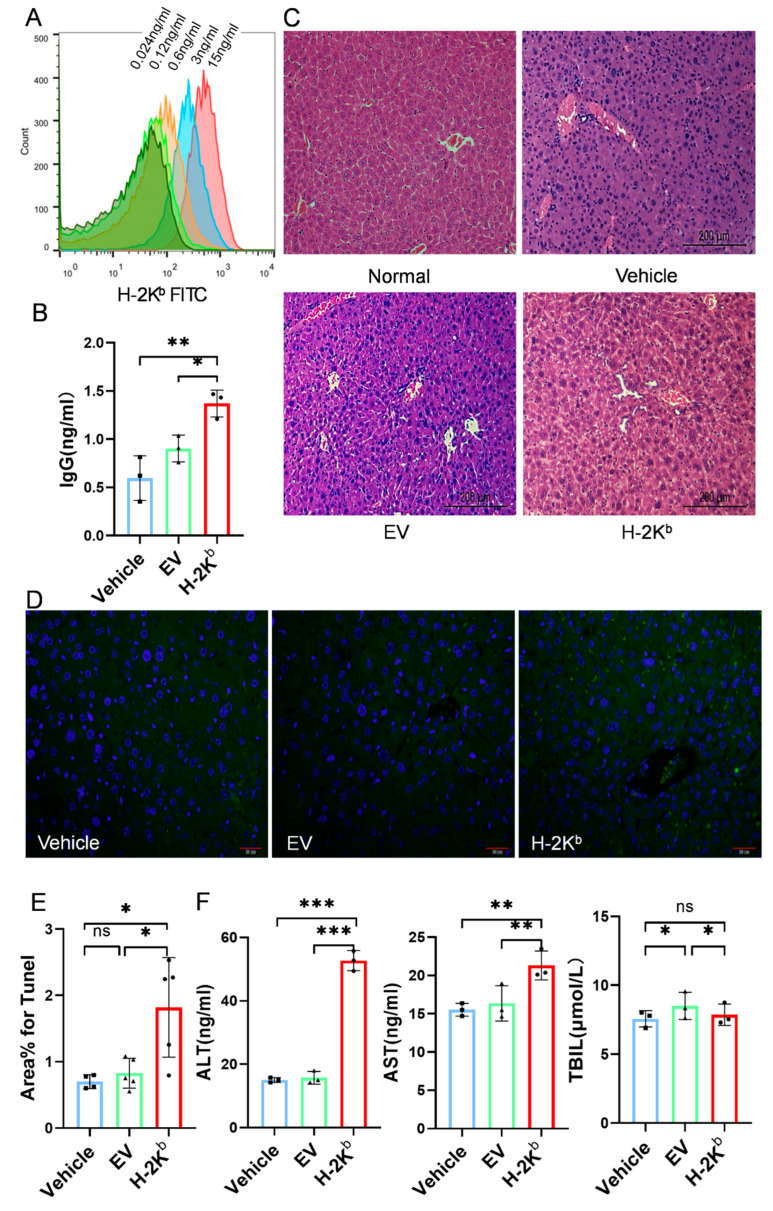
Serum anti-H-2K^b^ IgG and liver abnormalities following two intravenous injections of H-2K^b^-expressing ADSCs compared to empty vector-transduced ADSCs or vehicle only in a DBA/2J mouse model of diabetes and diabetic nephropathy: (**A**). Flow cytometry histogram overlays demonstrating sequentially higher fluorescence intensity of H-2K^b^-expressing ADSCs incubated with increasing concentrations of monoclonal anti-H-2K^b^ antibody and secondarily stained with anti-IgG-FITC antibody. (**B**). Graph depicting the result of a flow cytometry-based assay for quantification of anti-H-2K^b^ IgG antibodies in serum samples from three groups of diabetic DBA/2J mice that received two injections of either vehicle, empty-vector transduced ADCSs (EV) or H-2K^b^-expressing ADSCs (H-2K^b^). (**C**). Representative 100× photomicrographs of hematoxylin and eosin (H&E)-stained sections of liver tissue samples from non-diabetic DBA/2J mice (normal) and from diabetic DBA/2J mice that received two injections of either vehicle, empty-vector transduced ADCSs (EV) or H-2K^b^-expressing ADSCs (H-2K^b^). Cellular degeneration and inflammatory cell infiltration are evident in images from vehicle, EV and H-2K^b^ compared to normal, while disorganized hepatocyte arrangement, nuclear condensation and increased hepatocyte necrosis are most evident in the image from H-2Kb group. (**D**). Representative 200× fluorescence images of TUNEL-stained liver tissue samples from the same three groups of diabetic mice as for Panel (**B**). (**E**). Graph depicting the result of quantitative image analysis of the % positive TUNEL staining in liver tissue sections from the three groups of diabetic mice. (**F**). Graphs depicting the results of immunoassays of serum samples from the three groups of diabetic mice for the liver biomarkers ALT (left), AST (middle) and total bilirubin (TBIL, right). For column graphs, column heights represent group means, error bars represent SEM, and black dots represent results for individual samples. Statistical significance is indicated by ns = non-significant, * = *p* < 0.05, ** = *p* < 0.01, *** = *p* < 0.001.

To determine whether two sequential intravenous injections of H-2K^b^-expressing ADSCs also had immunogenic effects in diabetic DBA/2J mice, a flow cytometry-based assay was developed to detect IgG specific to H-2K^b^ in serum using C57BL/6 mouse splenocytes as targets. As shown in Figure 8A, serial dilutions of a monoclonal anti-H-2K^b^ IgG antibody were used to generate a standard curve based on MFI for this assay to allow for calculation of equivalent titers of anti-H-2K^b^ IgG in serum samples from experimental animals in each of the three diabetic groups (Figure 8B). This demonstrated significantly higher average titer in serum from the H-2K^b^-ADSC group compared to vehicle and EV-ADSC groups, consistent with an immunogenic response to the allo-antigen expressing ADSCs.

Histological analysis of pancreas and liver was also carried out by H&E staining of paraffin-embedded tissue sections from the three diabetic groups and from non-diabetic mice. The analysis of pancreas showed similar extent of islet destruction for mice from all three diabetic groups, suggesting that ADSCs administration did not result in pancreatic islet repair or regeneration. However, in liver sections, disruption of hepatocyte architecture with hepatocyte necrosis and inflammatory cell infiltration was observed to a greater extent in the H-2K^b^-ADSC group than in the vehicle and EV-ADSC groups which had milder hepatic abnormalities in comparison to liver sections from non-diabetic mice (Figure 8C). When apoptosis of liver cells was analyzed by TUNEL staining, the results indicated significantly higher numbers of apoptotic (TUNEL^+^) cells in liver sections from the H-2K^b^-ADSC group compared to vehicle and EV-ADSC groups (Figure 8D,E). Finally, in immunoassays to quantify three serum biomarkers of liver function, ALT and AST, but not TBIL, concentrations were significantly higher in the H-2K^b^-ADSC group—consistent with hepatocyte injury (Figure 8F).

Overall, these results indicated that, despite the beneficial effects for clinically relevant indices of diabetic nephropathy, two sequential intravenous injections of H-2K^b^-expressing ADSCs were also associated with induction of anti-H-2K^b^ IgG antibodies and with inflammatory liver damage in this model of diabetes that was not observed in recipients of fully autologous EV-ADSCs.

## 4. Discussion

The ongoing development of cell therapies, especially MSCs-based therapies, has brought hope of successful regenerative medicine approaches for people with DKD [6]. Compared with bone marrow, adipose tissue is relatively straightforward to obtain from both patients and healthy volunteers, and ADSCs have similar functions to other tissue-derived MSCs. This highlights the potential for ADSCs to be used as an autologous or allogeneic cell therapy. Using a genetic approach to express a single allogeneic MHC protein in otherwise autologous mouse ADSCs, our results provide new insights into the balance between the potential benefits and disadvantages of Allo- compared to Auto-ADSCs in a model of diabetes and DN.

In this study, male H-2K^d^ allotype DBA/2J mice, which have previously been reported to develop more severe DN than other mouse strains [18,19], were used to generate a model of DKD, and protocols for culture expansion and lentiviral transduction of ADSCs were developed [21]. During a 15-week experimental design, the effects of two sequential intravenous injections of ADSCs expressing the heterologous MHC class I molecule, H-2K^b^, were compared with those of fully autologous ADSCs and with no cell treatment. The results demonstrated that both H-2K^b^-ADSCs and EV-ADSCs reduced kidney/total body weight ratio, BUN and urine ACR, mesangial matrix expansion and glomerular/peri-glomerular fibrosis compared to vehicle alone without influencing glycemia and overall survival. Of these parameters, BUN and urine ACR were lower at the end of the experiment in mice that received H-2K^b^-ADSCs, suggesting a therapeutic advantage over fully autologous ADSCs. Furthermore, in a secondary experiment, H-2K^b^-ADSC administration was also associated with greater reductions in renal myeloid cell infiltration and collagen deposition. We also found evidence that repeated administration of H-2K^b^-ADSCs resulted in enhanced Treg systemically (in spleen) and locally (in kidneys) in diabetic animals—consistent with a recognized immune regulatory mechanism of action. Counteracting these potentially advantageous effects, however, we observed other immunological changes in recipients of llo-antigen-expressing ADSCs which may reflect detrimental effects. These included increased CD8/CD4 T cells ratios and increased DC and macrophage proportions in spleen; increased detectable circulating IgG antibodies against H-2K^b^; and histological and biochemical evidence of inflammatory liver injury. Importantly, by incorporating a control condition of DBA/2J ADSCs transduced with the empty lentiviral vector, we ruled out the possibility that some findings were due to lentiviral manipulation or other non-Allo-MHC I-mediated effects.

From a mechanistic perspective, our findings demonstrate that ADSC administration exerted clear anti-inflammatory and anti-fibrotic effects in the kidney. As there were no between-group differences in persistent hyperglycemia throughout the experiment, we can clearly conclude that these modulatory effects were not the result of increased repair of pancreatic islets. Of note, while intrarenal mRNA levels for TGF-β, TNF-α and IL-6 were similarly reduced in both EV-ADSC and H-2Kb-ADSC groups compared to vehicle-treated animals, other effects, including the reduction in CD11b^+^ myeloid cell infiltration and the reduction in Sirius Red-stained collagen deposition, were more potent in the presence of ADSC-delivered allo-antigen. Notably, mRNA expression of the Treg-specific transcription factor FOXP3 was significantly higher only in kidney tissue from the H-2K^b^-ADSC group. Furthermore, in our secondary experiment, this observation was strengthened by the finding of increased infiltration of FOXP3^+^ cells in the kidneys of H-2K^b^-ADSC-treated animals. This suggests that the additional beneficial effects of allo-antigen-expressing ADSCs in DN may be mediated by increasing the number of Treg within the kidneys. Mechanisms by which this effect could be induced by Allo-MSCs include a direct interaction between the MSCs and allo-antigen-reactive Treg or an indirect pathway of presentation of allo-antigen-derived peptides by recipient antigen-presenting cells which have been reprogrammed to pro-tolerogenic phenotypes while taking up MSCs-derived proteins [16,17]. An alternative potential anti-inflammatory mechanism of intravenous ADSCs is the enhancement of efferocytosis (re-programming of myeloid cells to anti-inflammatory/pro-regulatory phenotypes following phagocytosis of apoptotic cells), which has been documented as a mechanism of action of MSCs in inflammatory conditions [24]. However, whether Allo-MSCs induce more potent efferocytosis than Auto-MSCs has not, to our knowledge, been clearly determined and will require further investigation. It should be noted that our observations do not identify a specific primary mechanism of action of allo-antigen-expressing ADSCs in DN—a key issue for the successful clinical translation of MSCs and other disease-modulating cellular therapies. In this regard, a more detailed analysis of tissue-localized Treg numbers, phenotype and function in diabetic animals following repeated administration of allo-antigen-expressing ADSCs will be of high interest in future experimental work. Similarly, further investigation of the functional properties of macrophages and dendritic cells in the spleen and at other sites will help to determine whether the observed increases in these cells contribute to the beneficial or potentially detrimental immunological effects of allo-antigen-expressing MSCs.

T lymphocytes, including CD4^+^ and CD8^+^ effector T cells, play central roles in cellular and humoral adaptive immunity in vivo, and are known to be important mediators of autoimmunity in type 1 diabetes as well as of the severity of end-organ complications of both type 1 and type 2 diabetes, including DN [25,26]. Our results for systemic (splenic) T cell profiling of the H-2K^b^-ADSCs-treated and control groups of diabetic mice showed that H-2K^b^-ADSCs were associated with decreased proportions of CD4^+^ T cells, increased proportions of CD8^+^ T cells and, as a result, decreased CD4/CD8 T cell ratios. Along with this, EV-ADSCs and, to a greater extent, H-2K^b^-ADSCs were also associated with increased proportions of Treg among the splenic CD4^+^ T cells. Overall, the findings for splenic T cell subtypes are consistent with distinct modulatory effects of MSCs administration on systemic cellular immune responses in the setting of diabetes mellitus and DN, which are more prominent in the presence of MSCs-expressed allogeneic MHC protein. Concurrently, increased proportions of activated CD8^+^ T cells and antigen-presenting cells in the spleen, and higher serum IgG antibody against H-2K^b^ are likely to reflect activation of the host’s immune response to allo-MHC I. We interpret these findings as evidence of a dual immune response, wherein local tissue-protective effects coexist with systemic allo-MHC antigen recognition. This dichotomy highlights the complex immunological consequences of Allo-MSC administration and suggests that renal benefits may occur despite concurrent alloimmune activation. Further experimental studies will be necessary to explore the impact of these changes on systemic and localized inflammation and immune-mediated tissue injury in patients with both type 1 and type 2 diabetes.

In regard to the observed liver injury, it has been reported that MSCs accumulate in the lungs following injection into mice through the tail vein route, and then gradually migrate to other organs, including liver, within 10 days [27]. We and others have reported distribution of at least small proportions of intravenously administered MSCs to the liver, spleen and kidneys in mouse models of diabetes and other inflammatory disorders [21,28]. It is possible, therefore, that increased ADSC trapping and/or a localized allo-antigen-specific, re-call immune response to H-2K^b^-ADSCs occurred within the liver following the second cell injection. The fact that repeated administration was associated with detectable anti-H-2K^b^ IgG in serum is also in keeping with the induction of allo-antigen-specific immune response following primary and secondary exposure to H-2K^b^-ADSCs. Although there are differences in the expression of surface markers and immunosuppressive hyperglycemia between species, immune responses against MSCs-delivered allo-antigens have been observed in a variety of species, including rats, baboons, macaques, and pigs [17,29]. Thus, Allo-MSCs, while not as highly immunogenic as unmatched fibroblasts, splenocytes or hematopoietic stem cells, may also elicit humoral and cellular immune responses in vivo [16]. Importantly, however, such an adverse immunological effect has not been observed in human recipients of Allo-MSCs therapies, including in a recent trial of Allo-MSCs for DKD [13,30].

Some limitations of the current study must be acknowledged. The primary aim of the work was to evaluate host immune responses and renal outcomes following administration of either autologous or alloantigen-expressing ADSCs. Thus, experiments were not performed to characterize and compare the in vivo fates of EV- and H-2K^b^-ADSCs. As noted above, however, differences in the migration or persistence of Allo- and Auto-ADSCs could contribute to immunogenicity and therapeutic outcomes and should be addressed in future studies using cell-tracking techniques. Furthermore, as our approach does not fully recapitulate the complexity of clinically relevant allogeneic mismatches, our findings should be interpreted as reflecting one defined component of Allo-MSCs immunogenicity, rather than the entire clinical scenario. Nevertheless, MHC class I proteins represent a key component of allogeneic antigen recognition in clinical transplantation and cell therapy as they are expressed by essentially all transplanted cells. In addition to the potential for species-specific differences, it should also be noted that cell dosing per unit weight is typically much greater in rodent experiments compared to human trials. Despite this, our results do indicate that ongoing close attention must be paid to immunological responses and systemic effects of human subjects receiving investigational Allo-MSCs therapies—particularly if repeated dosing is planned. We also highlight that the statistical power of some of the analyses carried out at the terminal time-point of the experimental protocol was reduced by attrition stemming from the relative severity of the diabetes model, which likely limited our capacity to detect some between-group differences. Finally, we recognize that some findings of this study, which was performed only in male, DBA/2J mice, could be strain- or gender-dependent on the basis of STZ sensitivity or other genetically determined variations. Thus, future replication of these results in female animals and in other models of diabetes and DN will also be important.

Given the large amount of experimental evidence of disease-modulating effects of MSCs in diabetes and its major complications, successful advancement of this treatment approach to clinical practice has potential to improve the quality of life and reduce the very large burden of harm caused by this disease [6,31,32]. Our preclinical findings in this study emphasize the fact that, in order to maximize the future impact of Allo-MSC-based therapies on the burden of diseases such as DKD, it will be essential to develop Allo-MSC products with high disease-modulating capacity, excellent safety profile and affordable cost [33,34]. In future preclinical studies, several strategies could be explored to improve this balance, including modulation of dosing frequency and timing, reduction or transient expression of allo-antigens, and engineering approaches aimed at attenuating immunogenicity of Allo-MSCs while preserving therapeutic function. In addition, incorporating comprehensive immune monitoring and biodistribution analyses in future studies will be essential to better define safety windows and identify thresholds beyond which immunological activation outweighs therapeutic benefit. The frequency of administration of MSCs and other cell therapies will be much lower than that of pharmacological products, providing the benefit of increased compliance. It also provides a basis for ongoing research and innovation to develop optimized, cost-effective pipelines for cell manufacture, scale-up, transport and patient administration. Continued investigation of the comparative influences of Auto- and Allo-MSCs on Treg and other regulators of inflammation and immune response within the kidneys as well as on potentially detrimental immunological effects will increase the knowledge base for developing stem cell therapies. Identifying optimal processes for stem cell transduction may also bring economic benefits for companies developing novel cell and gene therapies.

## 5. Conclusions

Using a novel approach, our results provide new insights into the potential range of effects of two sequential intravenous administrations of Allo-MHC I-expressing MSCs in the setting of diabetes and DN. Of interest, we find that ADSCs transduced to express a specific allogeneic class I MHC protein exerted both beneficial and adverse effects compared to vehicle- and Auto-MSCs-treated controls. Overall, improvements in parameters of DN severity were most prominent in animal recipients of repeated doses of H-2K^b^-ADSCs, and these improvements were accompanied by modifications to the intra-renal and systemic immune cell repertoire as well as to intra-renal fibrosis. Nonetheless, they also resulted in evidence of inflammatory liver injury and detectable IgG response to the allogeneic MHC protein. These results highlight the need for continued investigation of the complex immunological effects of allo-antigen expressing MSCs in diabetes mellitus as a means to further enhance their efficacy for treating or preventing diabetic complications while avoiding potential adverse effects. Furthermore, as immunological responses in murine model systems such as those we describe here may have important differences to their counterparts in humans, it will also be necessary to conduct complementary mechanistic studies of MSCs-delivered allo-antigens involving human immune cells or humanized models [35].

## Data Availability

The original contributions presented in this study are included in the article. Further inquiries can be directed to the corresponding authors.
